# A phase I/II study of nintedanib and capecitabine for refractory metastatic colorectal cancer

**DOI:** 10.1093/jncics/pkae017

**Published:** 2024-05-03

**Authors:** Patrick M Boland, John M L Ebos, Kristopher Attwood, Michalis Mastri, Christos Fountzilas, Renuka V Iyer, Christopher Banker, Andrew K L Goey, Robert Bies, Wen Wee Ma, Marwan Fakih

**Affiliations:** Department of Medical Oncology, Rutgers Cancer Institute of New Jersey, New Brunswick, NJ, USA; Department of Medicine, Roswell Park Comprehensive Cancer Center, Buffalo, NY, USA; Department of Cancer Genetics and Genomics, Roswell Park Comprehensive Cancer Center, Buffalo, NY, USA; Department of Biostatistics and Bioinformatics, Roswell Park Comprehensive Cancer Center, Buffalo, NY, USA; Department of Cancer Genetics and Genomics, Roswell Park Comprehensive Cancer Center, Buffalo, NY, USA; Department of Medicine, Roswell Park Comprehensive Cancer Center, Buffalo, NY, USA; Department of Medicine, Roswell Park Comprehensive Cancer Center, Buffalo, NY, USA; Department of Pharmacology and Therapeutics, Roswell Park Comprehensive Cancer Center, Buffalo, NY, USA; Department of Pharmacology and Therapeutics, Roswell Park Comprehensive Cancer Center, Buffalo, NY, USA; Department of Pharmaceutical Sciences, School of Pharmacy and Pharmaceutical Sciences, University at Buffalo, Buffalo, NY, USA; Department of Hematology and Oncology, Taussig Cancer Institute, Cleveland Clinic, Cleveland, OH, USA; Department of Medicine, City of Hope Comprehensive Cancer Center, Duarte, CA, USA

## Abstract

**Background:**

Nintedanib is a tyrosine kinase inhibitor with efficacy in bevacizumab-resistant colorectal cancer models. This phase I/II study evaluated the recommended phase II dose and efficacy of nintedanib and capecitabine in refractory metastatic colorectal cancer.

**Methods:**

Key eligibility criteria included refractory metastatic colorectal cancer and ECOG performance status of 1 or lower. The primary endpoint was 18-week progression-free survival (PFS). A 1-sided binomial test (at α = .1) compared the observed 18-week PFS with a historic control of .25.

**Results:**

Forty-two patients were enrolled, including 39 at the recommended phase II dose. The recommended phase II dose was established to be nintedanib 200 mg by mouth twice daily and capecitabine 1000 mg/m^2^ by mouth twice daily. The protocol was evaluated for efficacy in 36 patients. The 18-week PFS was 42% (15/36 patients; *P* = .0209). Median PFS was 3.4 mo. Median overall survival was 8.9 mo. Sixteen (44%) patients experienced a grade 3/4 adverse event, most commonly fatigue (8%), palmoplantar erythrodysesthesia (8%), aspartate aminotransferase elevation (6%), asthenia (6%), pulmonary embolus (6%), and dehydration (6%). Osteopontin levels at cycle 1, day 1 and cycle 3, day 1 as well as ΔCCL2 levels correlated to disease control at 18 weeks.

**Conclusions:**

The combination of nintedanib and capecitabine is well tolerated. Clinical efficacy appears to be superior to regorafenib or tipiracil hydrochloride monotherapy. Further investigation of similar combinations is warranted.

**ClinicalTrials.gov identifier:**

NCT02393755

Despite recent advances in the detection and treatment of metastatic colorectal cancer (CRC), the 5-year overall survival stands at less than 15% (1). In the refractory setting, survival remains 5 to 7 months after failure of the classic chemotherapeutics: fluoropyrimidines, oxaliplatin, irinotecan, bevacizumab, and anti–epidermal growth factor receptor targeted therapies (2,3). Additional oral agents, including regorafenib and tipiracil hydrochloride (TAS-102), are approved in this setting, both of which modestly improve outcomes through disease stabilization. Median progression-free survival (PFS) for both drugs is approximately 2 months; in the respective pivotal studies, regorafenib and TAS-102 improved median overall survival by less than 2 months (2,3).

Nintedanib (BIBF1120) is a small molecule tyrosine kinase inhibitor of vascular endothelial growth factor (VEGF) receptors 1, 2, and 3; fibroblast growth factor receptors 1, 2, and 3; and platelet-derived growth factor receptor α/β, with activity against Flt-3, RET, Src, Lck, and Lyn (4). Nintedanib demonstrated significant tumor growth inhibition in multiple models, with marked reduction in tumor vessel density (4). In humans, maximum plasma concentrations occur at 2 to 4 hours, reaching terminal half-life within 7 to 19 hours (5). In phase I investigation, nintedanib monotherapy elicited responses and provided disease control (6). Combinations with chemotherapy have proven tolerable, in contrast to many prior tyrosine kinas inhibitor–chemotherapy combinations (7). A phase II study that paired leucovorin calcium, fluorouracil, and oxaliplatin with either nintedanib or bevacizumab demonstrated similar rates of grade 3 or higher adverse events in both study arms (8).

We conducted a phase I/II study of nintedanib and capecitabine for individuals with refractory metastatic CRC. Study objectives were to 1) establish safe dosing for the combination, 2) assess whether the regimen improved PFS over historic comparators (regorafenib and TAS-102), and 3) examine whether plasma protein analysis may uncover 1 or more biomarkers predictive of toxicity or treatment efficacy.

## Methods

### Overall study design

This study was a multicenter, single-arm trial of capecitabine and nintedanib in patients with refractory metastatic CRC (ClinicalTrials.gov identifier: NCT02393755). The study was performed in accordance with the Declaration of Helsinki and the principles of Good Clinical Practice. The protocol was approved by the institutional review boards at Roswell Park Comprehensive Cancer Center (Buffalo, NY) and City of Hope Comprehensive Cancer Center (Duarte, CA), and all patients provided written informed consent before undergoing study procedures.

### Patient eligibility

Eligible patients were 18 years of age or older and had an ECOG performance status of 0 to 1 and pathologically proven colorectal adenocarcinoma. Patients must have had intolerance to or progression after standard therapies: a fluoropyrimidine, oxaliplatin, irinotecan (and for patients with *RAS* wild-type disease), an anti–epidermal growth factor receptor–based therapy. Measurable disease per Response Evaluation Criteria in Solid Tumors (RECIST), version 1.1, was required. Bilirubin was required to be at or below the upper limit of normal (ULN), with aspartate aminotransferase (AST) and alanine aminotransferase (ALT) at or below 1.5 × ULN if without liver metastases and at or below 2.5 × ULN if with liver metastases. Creatinine was required to be at or below 1.5 × ULN with creatinine clearance above 50 mL/min by Cockcroft-Gault equation. Prior treatment with nintedanib or regorafenib and the presence of conditions that raised the risk of receiving anti-VEGF receptor therapy were exclusionary. Patients with prior intolerance to capecitabine were excluded, including those with prior grade 3 palmoplantar erythrodysesthesia or diarrhea.

### Study objectives

The primary objectives of the phase I study were to examine the dose-limiting toxicities of nintedanib when combined with capecitabine and estimate the maximum tolerated dose for establishment of the recommended phase II dose. The primary objective of the phase II expansion was to estimate the PFS of nintedanib and capecitabine at 18 weeks via RECIST, version 1.1, criteria. Secondary objectives were to assess the median PFS, the median overall survival, and the objective response rate in patients treated at the recommended phase II dose. Survival times were defined from the start of protocol treatment. Assessment of the toxicity and overall tolerability using the National Cancer Institute Cancer Therapy Evaluation Program Common Terminology Criteria for Adverse Events, version 4.0, was an additional aim. Exploratory analysis included measurement of circulating angiogenic cytokines, measurement of nintedanib drug levels, and pharmacokinetic modeling.

### Treatment

Treatment was administered in 21-day cycles. Nintedanib was taken orally twice daily at a dose of 150 (dose level 1) or 200 mg (dose level 2) throughout the treatment period. At both dose levels, capecitabine was administered orally at a daily dose of 2000 mg/m^2^, administered as 2 divided doses (approximately 1000 mg/m^2^ per dose) and taken from days 1 through 14 of every cycle. Both medications were to be separated by 12 hours (SD = 2 hours) from the prior dose and taken within 30 minutes of a meal.

### Study endpoints

The primary study endpoint for the phase I study was establishment of the recommended phase II dose. The primary endpoint of the phase II study was to determine PFS at 18 weeks based on RECIST, version 1.1, criteria. Secondary endpoints were estimation of median PFS, median overall survival, objective response rate, and aggregate rates of adverse events by Common Terminology Criteria for Adverse Events, version 4.0, criteria in patients treated at the recommended phase II dose.

### Study assessments

The dose-limiting toxicity evaluation period was defined as the first 21 days of therapy (1 cycle). A dose-limiting toxicity was defined as any of the following deemed to be at least possibly drug-related: grade 3 or higher nonhematologic toxicity (except transient electrolyte abnormality, alopecia, suboptimally treated nausea, vomiting or diarrhea, or isolated elevated of γ-glutamyl transpeptidase); nintedanib-related hepatotoxicity additionally qualified as a dose-limiting toxicity (AST or ALT above 5 × ULN, independent of bilirubin, or AST or ALT above 2.5 × ULN together with total bilirubin above 1.5 × ULN); grade 4 or higher neutropenia lasting longer than 7 days; febrile neutropenia; grade 4 thrombocytopenia; or grade 3 thrombocytopenia associated with bleeding or requiring transfusions. The inability to resume nintedanib dosing within 14 days of stopping because of an adverse event was also considered a dose-limiting toxicity. (See the supplemental materials, available online, for pharmacokinetic and biomarker methodologies.)

### Statistical considerations and analysis

#### Phase I

The phase I portion of the study began by enrolling 3 patients at dose level 1, and dose escalation followed the standard 3 + 3 decision rules. The recommended phase II dose was defined as the maximum dose level at which 1 or fewer dose-limiting toxicities were observed in 6 patients. The 6 phase 1 patients treated at the recommended phase II dose are included in stage 1 of the phase II study.

#### Phase II

The primary objective of the phase II study was to evaluate the 18-week PFS rate in patients treated at the recommended phase II dose. The primary outcome was the 18-week PFS status, where patients whose disease progressed or who discontinued treatment for disease-related causes before 18 weeks were considered “failures” and those who were disease progression free at 18 weeks were considered “successes.” Patients who did not complete at least 14 days of treatment either because of toxicity or non–disease-related factors were considered nonevaluable and replaced. Historically, the 18-week PFS rate is 5% and 25% for patients with refractory metastatic CRC treated with placebo and regorafenib, respectively. Therefore, we used a 1-sided binomial exact test to evaluate the following hypotheses: H_0_: π = 0.25 vs H_A_: π > 0.25, where π is the true 18-week PFS rate. The 18-week PFS rate was also estimated using an 80% confidence interval (CI) obtained using the Pearson-Clopper method. The combination of nintedanib and capecitabine was expected to provide an 18-week PFS of at least 0.40 in patients with refractory metastatic CRC. This single-stage design required 36 evaluable patients to achieve 80% power at a significance level of .10. The sample size was calculated using PASS, version 11, statistical software (NCSS Statistical Software, Kaysville, UT).

Adverse events were summarized by grade using frequencies and relative frequencies. Overall survival and PFS were summarized using standard Kaplan-Meier methods, with estimates of the median survival obtained with 90% confidence intervals. Response was summarized using frequencies and relative frequencies. Only patients who completed at least 2 cycles of therapy were deemed evaluable for response assessment.

### Exploratory biomarker analyses

In patients with at least 1 predose; cycle 1, day 15; or cycle 3, day 1 observation, the log of each biomarker was modeled as a function of time-point and random subject effect (with an autoregressive covariance structure) using a linear mixed model. The mean levels were compared between predose and both cycle 1, day 15 and cycle 3, dose 1 using Dunnett-adjusted tests about the least square means. All model assumptions were verified graphically.

The Spearman correlation coefficient was used to evaluate the relationship between baseline biomarker levels and changes in tumor burden. Associations between biomarkers and both response and survival outcomes were evaluated using logistic and Cox proportional hazards models, respectively. Models were fit using the Firth method, and model assumptions were verified graphically. Odds ratios (ORs) or hazard ratios (HRs) were obtained from model estimates and reported with 90% confidence intervals.

All analyses were conducted in SAS, version 9.4, statistical software (SAS Institute Inc, Cary, NC) at a significance level of .10.

## Results

### Patient characteristics

Forty-two patients were enrolled between May 8, 2015, and July 17, 2017. Twenty-three (54.8%) patients had an ECOG performance status of 0, and the remainder had an ECOG performance status of 1. Thirty (71%) patients had *KRAS* or *NRAS* mutated tumors. See Table 1 for additional details.

**Table 1.** Baseline demographics (n = 42)

Characteristic	Value
Sex, No. (%)	
Female	24 (57)
Male	18 (43)
Age, mean (range)	58 (36-78)
Age <65 y, No. (%)	32 (76)
Age ≥65 y, No. (%)	10 (24)
Race, No. (%)	
Asian	5 (12)
Black	1 (2)
Other/not specified	3 (7)
White	33 (79)
ECOG performance status, No. (%)	
0	23 (55)
1	19 (45)
Body weight, mean (range), kg	77.2 (41.1-126.4)
Mutational Status	
*RAS* mutant	30 (71)
*RAS* wild type	9 (21)
Not available	3 (7)

### Treatment exposure and dose-limiting toxicities

Nine patients were enrolled in the phase I dose-escalation study, 3 at dose level 1 and 6 at dose level 2. Thirty-three additional patients were enrolled in the phase II study. As no dose-limiting toxicities were observed, dose level 2 was selected for the phase II study: nintedanib 200 mg orally twice daily and capecitabine 2000 mg/m^2^ in split doses, administered at 14 days of a 21-day cycle.

At the time of analysis, all patients had discontinued therapy. The median number of treatment days was 75. The most common reason for discontinuation was disease progression (83.3% [n = 35]). One patient discontinued because of unacceptable toxicity, and 2 patients (4.8%) withdrew consent. Three patients treated at the recommended phase II dose discontinued treatment within 2 weeks and were replaced; withdrawal was related to capecitabine-induced coronary vasospasm, a more-than-2-week treatment delay because of a serious adverse event, and elective surgery.

### Efficacy

**Figure 1.**
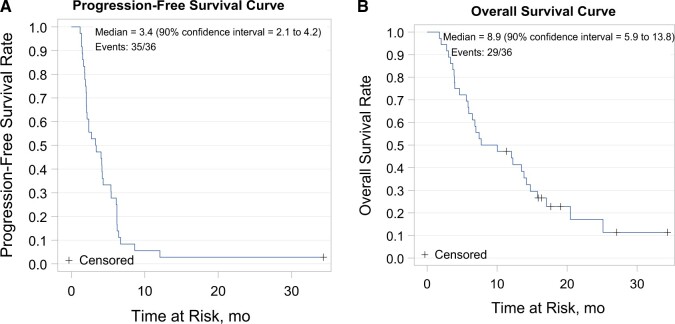
Progression-free survival (**A**) and overall survival (**B**) in patients treated at the recommended phase II dose.

At the final data cutoff, median follow-up was 19.1 months (range = 1.7-34.3). The 18-week investigator-assessed PFS was 41.7%, (80% CI = 31.7% to 52.3%, *P* = .0209), which was improved compared with the historic control (Figure 1, A). The median PFS was 3.4 months (90% CI = 2.1 to 4.2). Median overall survival was 8.9 months (90% CI = 5.9 to 13.8), with 29 of 36 events documented at data cutoff (Figure 1, B). No objective responses were observed. Twenty-one patients (58%) achieved stable disease as the best response, whereas 42% experienced progressive disease. In a post hoc analysis, a notable fraction of the 42 patients treated on study had prolonged disease control on therapy: 9 (21.4%) were on treatment for at least 24 weeks, and 2 (4.8%) were on treatment for at least 36 weeks.

**Figure 2.**
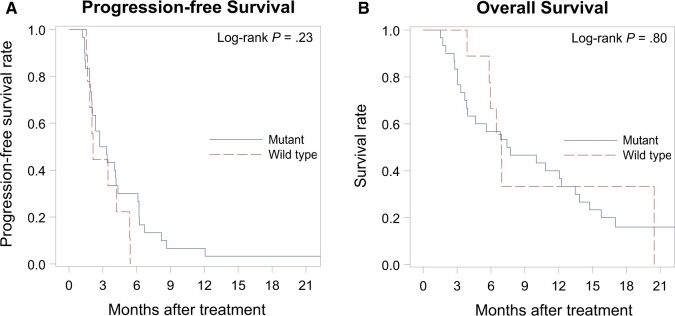
Progression-free survival (**A**) and overall survival (**B**), by *RAS* status.

Outcomes were analyzed by *RAS* status, without significant differences (*P* = .80 for overall survival and *P* = .23 for PFS). Median overall survival for mutated vs wild-type tumors was 7.6 (95% CI = 3.8 to 13.5) and 6.9 (95% CI = 3.9 to 20.5) months, respectively (Figure 2, A). Median PFS for mutated vs wild type was 3.0 (95% CI = 2.0 to 4.3) and 2.1 (95% CI = 1.5 to 5.4) months, respectively (Figure 2, B).

### Safety: treatment-emergent serious adverse events and adverse events

Overall, 100% of the 39 patients at the recommended phase II dose experienced treatment-related adverse events. Seventeen (44%) patients experienced treatment-related grade 3 or greater adverse events, including 2 (5%) grade 4 adverse events: dehydration and lymphopenia. The most common grade 3 or greater adverse events were fatigue (8%), palmoplantar erythrodysesthesia (8%), nausea (5%), dehydration (5%), and pulmonary embolism (5%). The most common treatment-related adverse events occurring in 10% or more of patients were nausea (62%), fatigue (56%), diarrhea (56%), vomiting (54%), AST elevations (36%), anorexia (36%), palmoplantar erythrodysesthesia (36%), and ALT elevations (33%). Most adverse events were grade 1 or grade 2 and readily reversible with dose interruptions or reductions. Twenty-one (50%) of the 42 patients treated on study required a dose reduction because of toxicity, including 19 (49%) of 39 treated at the recommended phase II dose. Treatment-related adverse events occurring in 10% or more of patients or otherwise of special interest are depicted in Table 2.

**Table 2.** Treatment-related adverse events in 10% or more of patients and of special interest

Adverse event	Any grade, %	Grade 2, %	Grade 3, %	Grade 4, %
Any adverse event	97	41	38	5
**General/constitutional**				
Fatigue	56	26	8	0
Decreased appetite	36	5	3	0
Dysgeusia	10	0	0	0
Weight loss	13	5	0	0
Dehydration	10	5	3	3
Dizziness	10	0	0	0
Headache	10	0	0	0
Peripheral neuropathy	10	3	0	0
**Gastrointestinal disorders**				
Diarrhea	56	10	3	0
Nausea	62	15	5	0
Vomiting	54	5	3	0
Stomatitis	13	3	0	0
Abdominal pain	10	3	3	0
Constipation	10	3	0	0
**Laboratory investigations**				
Aspartate aminotransferase increase	36	5	5	0
Alanine aminotransferase increase	33	5	3	0
Alkaline phosphatase increase	23	5	0	0
Bilirubin increase	15	3	3	0
White blood cell count decreased	5	0	0	0
Neutrophils decreased	3	0	3	0
Anemia	18	3	3	0
Platelets decreased	5	0	0	0
**Vascular disorders**				
Pulmonary embolism	5	0	5	0
Deep vein thrombosis	3	0	3	0
Hemoptysis	3	0	0	0
Hypertension	13	8	3	0
**Skin and subcutaneous disorders**				
Dry skin	18	3	0	0
Palmoplantar erythrodysesthesia	36	18	8	0

### Pharmacokinetics

Primary and secondary pharmacokinetic parameters were estimated using a 1-compartment disposition model with first-order absorption and elimination (Supplementary Materials—Population Pharmacokinetic Modeling Results, Supplementary Tables 1-4, available online).

The mean (SD) time to reach maximum plasma concentration was 3.7 (2.3) hours on day 1 and 3.1 (1.4) hours on day 15. The mean (SD) maximum plasma concentration was 48.7 (45.6) ng/mL on day 1 and 42.9 (30.6) ng/mL on day 15. The mean (SD) area under the curve (AUC) on day 1 was 494.8 (512.5) ng×h/mL and on day 15 was 334.4 (173.1) ng×h/mL. There was no association between any pharmacokinetic parameter and PFS or overall survival, nor was there a significant difference in outcome when comparing the stratum with greater drug exposures with the stratum with lower plasma drug levels.

Maximum plasma concentration at cycle 1, day 15 was associated with the degree of plasma CCL2 change (increase) from cycle 1 to cycle 3 (correlation coefficient = 0.53684, *P* = .0147). The AUC at cycle 1, day 15 was also associated with plasma CCL2 change (increase) from cycle 1 to cycle 3 (correlation coefficient = 0.59850, *P* = .0053). No additional biomarkers demonstrated a significant relationship to the nintedanib maximum plasma concentration or AUC.

### Circulating angiogenic factors

Of the 8 circulating angiogenic factors analyzed, only modulation of VEGF receptor 2 levels were statistically significant, decreased at both cycle 1, day 15 (*P* = .097) and cycle 3, day 1 (*P* < .001) compared with baseline (Table 3). Pretreatment plasma osteopontin levels were associated with probability of achieving the 18-week PFS outcome (odds ratio [OR] = 0.976, *P* = .0481), as were pretreatment CCL2 levels (OR = 1.011, *P* = .0742) and SCF levels (OR = 1.055, *P* = .538). CCL2 increase from cycle 1, day 1 to cycle 3, day 1 was significantly associated with 18-week PFS status (OR = 0.982, *P* = .0473), with increasing levels linked to increased odds of disease progression.

**Table 3.** Modulation in circulating angiogenic factor levels during treatment^a^

Marker	Least square mean (SE)			Dunnett-adjusted *P*	
	Predose	Cycle 1, day 15	Cycle 3, day 1	Cycle 1, day 15 vs predose	Cycle 3, day 1 vs predose
VEGF receptor 2	18 394.53 (1826.80)	15 994.69 (1835.11)	14 438.33 (1854.49)	.097	<.001
Interleukin 8	22.44 (5.69)	21.89 (5.73)	19.94 (5.69)	.66	.86
CCL2	287.02 (36.62)	287.28 (36.62)	263.29 (37.17)	.58	.18
VEGF	40.30 (6.80)	52.72 (6.62)	40.77 (6.90)	.26	1.00
SCF	73.49 (6.73)	72.65 (6.73)	71.26 (6.75)	.58	.36
OPN	86 695.45 (9071.36)	78 409.36 (9182.26)	81 202.36 (9282.67)	.25	.54
Leptin	14 522.82 (2966.78)	14 019.20 (2983.93)	13 041.96 (2992.98)	.98	.25
Placental growth factor	4.70 (0.89)	6.35 (0.83)	5.44 (0.87)	.14	.60

a VEGF = vascular endothelial growth factor.

**Figure 3.**
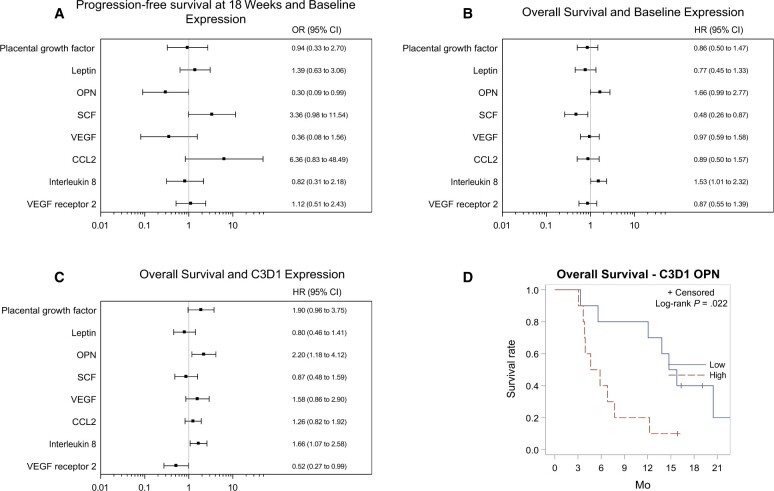
Outcomes by circulating angiogenic factor levels. Circulating angiogenic factor levels and relationship of (**A**) baseline levels to 18-week progression-free survival status, (**B**) baseline levels to overall survival, (**C**) C3D1 levels to overall survival, and (**D**) overall survival is improved in patients with OPN levels below median (low) vs above median (high). CI = confidence interval; HR = hazard ratio; OR = odds ratio; VEGF = vascular endothelial growth factor.

We also analyzed biomarker associations with best response (complete response, partial response, and stable disease vs progressive disease). Osteopontin level before treatment (OR = 0.974, *P* = .675) as well as at cycle 3, day 1 (OR = 0.963, *P* = .0552) was significantly correlated to the probability of achieving stable disease (Figure 3). Concerning the additional circulating angiogenic factors, pretreatment levels, on-treatment levels, and modulation of levels from baseline were not associated with probability of achieving disease stability.

**Figure 4.**
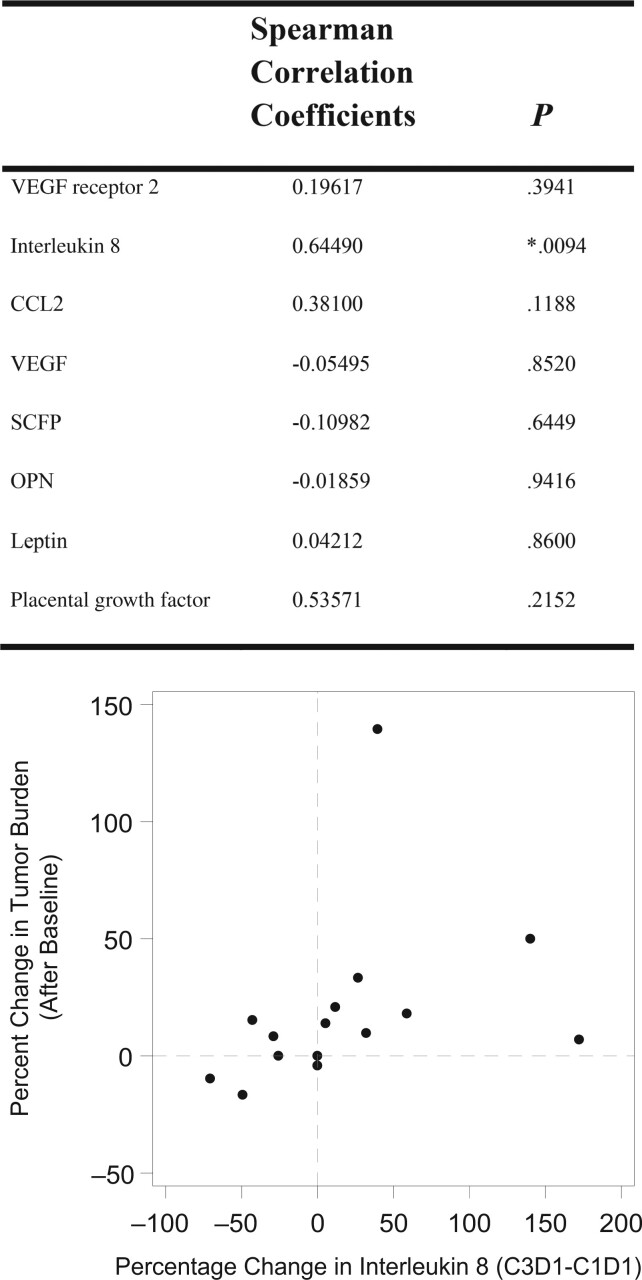
Correlation between change in tumor burden and change in biomarkers from cycle 1, day 1 to cycle 3, day 1. VEGF = vascular endothelial growth factor

Overall survival was significantly correlated to plasma osteopontin levels at baseline (HR = 1.661, *P* = .0523) and at cycle 3, day 1 (HR = 2.203, *P* = .0136). Survival and interleukin 8 levels were similarly associated before treatment (HR = 1.523, *P* = .0452) and at cycle 3, day 1 (HR = 1.659, *P* = .0232). Finally, although baseline VEGF receptor 2 had no relation, VEGF receptor 2 level at cycle 3, day 1 correlated with overall survival (HR = 0.912, *P* = .0454), as were the degree of VEGF receptor 2 modulation between cycles 3 and 1. Similarly, cycle 3 placental growth factor level but not baseline placental growth factor level correlated with overall survival (HR = 1.61, *P* = .0637), as did change in placental growth factor (HR = 0.531, *P* = .0355). Of note, there was a correlation between percentage change in tumor burden and change in interleukin 8 levels from before treatment to cycle 3, day 1 (Spearman correlation coefficient = 0.67889, *P* = .0054). Using analysis of covariance models, this association was maintained when adjusting for baseline tumor target volume size (Figure 4).

## Discussion

Antiangiogenic agents have proven survival benefit when used across multiple lines of therapy (9). At the time this study was designed, prior small investigations of antiangiogenic therapy combined with fluoropyrimidines in the refractory setting had demonstrated clinical efficacy. The National Cancer Institute TRC-0301 study employed infused 5-fluorouracil and bevacizumab, achieving a median PFS of 3.5 months and an overall survival of 9 months (10). These results were replicated in a separate study of this regimen, yielding a median PFS of 3.5 months and an overall survival of 7.7 months (11). A third investigation paired the antiangiogenic tyrosine kinase inhibitor sunitinib with capecitabine and achieved a median PFS of 137 days (4.6 months) and a median overall survival of 291 days (9.7 months) (12). In the CORRECT and RECOURSE trials, median PFS was approximately 2 months and overall survival at 6.4 and 7.1 months for regorafenib and TAS-102, respectively (2,3). On this basis, we hypothesized that the oral combination regimen of nintedanib and capecitabine would improve outcomes over these historic standards.

Our clinical study met the primary endpoint of 18 week-PFS, supporting the activity of nintedanib and capecitabine in metastatic CRC. The historic control was extrapolated from the CORRECT and RECOURSE trials, where the 18-week PFS was approximately 25% (2,3). Our novel combination achieved a superior 18-week PFS rate of 41.7% (80% CI = 32% to 52%, *P* = .0209), a median PFS of 3.4 months, and an overall survival of 8.9 months, all supporting improved clinical activity. Since the proposal of this study, multiple additional trials of antiangiogenic agents and antimetabolites have been conducted in the refractory setting, with consistent results. The single arm N-TASK FORCE and EPOC1410 studies tested TAS-102 with the partners nintedanib and bevacizumab, respectively (13). These both achieved a median PFS of 3.7 months and a median overall survival of 9.2 and 11.4, respectively. A subsequent randomized phase II study additionally suggested the superiority of TAS-102 and bevacizumab over TAS-102 monotherapy (14). Most recently, the randomized phase III SUNLIGHT trial definitively established the superiority of TAS-102 plus bevacizumab over TAS-102 monotherapy, creating a new standard in this setting. In this study, continuation of antiangiogenic therapy improved median PFS from 2.4 to 5.6 months (HR = 0.44) and the overall survival from 7.5 to 10.8 months (HR = 0.61) (15).

Unfortunately, before the completion of our trial, further efforts to develop nintedanib in CRC were abandoned based on the placebo-controlled LUME-Colon 1 trial. This study demonstrated that in refractory metastatic CRC, nintedanib significantly increases PFS (HR = 0.58, *P* < .0001) but not overall survival compared with placebo (6.4 vs 6 months; HR = 1.01, *P* = .8659) (16). Thus, our results will not be directly translated to a follow-up trial. Multiple VEGF receptor–positive targeting tyrosine kinase inhibitors remain under active investigation, however. In the refractory metastatic CRC setting, fruquintinib represents a potent inhibitor of VEGF receptors 1, 2, and 3, which improved PFS and overall survival in the FRESCO and FRESCO-2 studies (17,18). Thus, related combinations with fruquintinib might hold promise.

Correlative pharmacokinetic studies suggested no alteration in nintedanib exposure with capecitabine co-administration. Drug levels were not linked to outcome, but nintedanib AUC at day 15 correlated with increased CCL2 levels, which in turn were associated with a greater likelihood of progressing before the 18-week time point. This finding is consistent with prior in vivo work that suggested the potential of bevacizumab to increase CCL2/MCP-1 levels (19). CCL2 is a key chemoattractant for tumor-associated macrophages, promoting vascularization, with prior links to CRC progression and antiangiogenic therapeutic resistance (20). Preclinical models suggest that blockade of CCL2 improves tumor control and suppresses tumor-associated macrophage accumulation (19). Our trial did not assess tumor microenvironment components, but these potential compensatory changes support further exploration of combined CCL2/CCR2 and angiogenic inhibition (21).

OPN was the circulating angiogenic factor most consistently linked to outcome. OPN is an extracellular matrix phosphoglycoprotein that binds to CD44-family receptors and α_v_β integrins (22). It is derived both from tumor cells and nonmalignant host sources, particularly myeloid cells (23). Elevated OPN levels have been linked to worse prognosis in patients with metastatic CRC treated with bevacizumab-based regimens as well as in other malignancies (24-26). Lower baseline OPN levels correlated with lack of progression at 18 weeks, with overall survival linked to lower baseline and cycle 3, day 1 levels. Although a trend emerged toward OPN decrease on treatment, it did not reach levels of significance. Prior data with another tyrosine kinase inhibitor, sunitinib, in murine models suggested the modulation of OPN levels to be a tumor-independent, drug-related, host-related effect of nintedanib (27). Of relevance, host-derived OPN appears to elaborate the immunosuppressive metastatic niche in multiple cancers (28-30). Further investigation into the role and potential modulation of OPN is merited.

Although the primary outcome of this study was reached, this was a relatively small phase II study without a randomized comparator. There is a potential that our finding of improved PFS at 18 weeks is simply due to chance, due to a small benefit derived from continuation of capecitabine in the refractory setting, or due to enrollment of a nonrepresentative population with a more indolent disease course. Some significant associations are highlighted from the biomarker studies, though these remain exploratory studies, subject to type I error from multiple-hypothesis testing. At the same time, the overall numbers were small and potentially underpowered to elucidate some relationships.

In conclusion, nintedanib plus capecitabine has encouraging clinical activity with a favorable toxicity profile. Additional randomized investigations of antiangiogenic and fluoropyrimidine combinations are merited in the refractory setting.

## Supplementary Material

pkae017_Supplementary_Data

## Data Availability

The data underlying this article are available in the article and in its online supplementary material.
