# Supplementary material for: A phase I/II study of nintedanib and capecitabine for refractory metastatic colorectal cancer
Source: JNCI Cancer Spectr. 2024 May 3;8(3):pkae017. doi: 10.1093/jncics/pkae017 (PMC11065487; doi:10.1093/jncics/pkae017)
Supplement: pkae017_Supplementary_Data [file pkae017_supplementary_data.pdf]

Supplementary Materials

Population Pharmacokinetic Model Development

Population pharmacokinetic (PK) modeling was performed using NONMEM 7.4.1 (ICON Development Solutions, Hanover, MD, USA) interfaced with PsN 4.8.1 (psn.sourceforge.net) and Pirana 2.9.7 (pirana-software.com). The model was developed utilizing PK data for nintedanib from the first 30 subjects (12 subjects from City of Hope Comprehensive Cancer Center and 18 subjects from Roswell Park Comprehensive Cancer Center). **Supplementary Table 1** provides the number of PK observations and the number of observations below the limit of quantitation (BLQ, 0.500 ng/mL) that were incorporated into the model. In total, 19 post-dose samples were BLQ. These BLQ observations were included in the analyses and treated as censored information using the M3 method (1). Briefly, this method simultaneously models continuous (quantifiable) data and categorical (BLQ) data, and this approach has been shown to minimize bias in parameter estimates compared to other approaches for handling BLQ data (2). All Cycle 1 - Day 1 pre-dose samples were BLQ, and these observations were not included in the PK analyses.

Conditional weighted residuals (CWRES) and individual weighted residuals (IWRES) were examined for identification of potential PK outliers that deviated significantly from the expected patterns in the concentration–time profiles. A threshold of |CWRES| and/or |IWRES| > 6 was applied prospectively for identification of suspect observations that would warrant investigation and potential exclusion from PK analysis. In the present analysis, none of the observations met this outlier criterion.

Population parameters were estimated using the first-order conditional estimation method with interaction with the Laplacian option specified. Throughout model development, nested models were compared using the objective function value (OFV), and standard diagnostic plots were generated to evaluate model fit (3).

Exponential functions were utilized to incorporate between-subject and between-occasion variability in PK parameters. Between-subject variability was included on all PK parameters for which it could be reliably estimated. Additive, proportional, and combined additive and proportional functions were considered for characterization of residual unexplained variability in drug concentrations (3).

Since this was an analysis using a preliminary dataset, subject characteristics that might contribute to between-subject variability in PK were not considered for inclusion in the model.

Population Pharmacokinetic Modeling Results

PK parameter estimates are provided in **Supplementary Table 2**. A one-compartment disposition model with first-order absorption and elimination was used to fit the PK data. Between-subject variability could only be estimated reliably for the first-order absorption rate constant. Cycle 1 - Days 1–2 and Cycle 1 - Day 15 were considered to be two separate PK occasions, and between-occasion variability in PK was observed to be high within many subjects. Therefore, between-occasion variability in bioavailability was incorporated into the model. A combined additive and proportional function best characterized the residual unexplained variability in nintedanib concentrations. Individual PK parameter estimates are provided in **Supplementary Table 3**.

Secondary PK parameter values (exposure estimates) are reported in **Supplementary Table 4**. Observed values for maximum plasma concentrations (C_{max}) and time to reach C_{max} (t_{max}) on Days 1 and 15 are also shown. The area under the concentration–time curve from 0 to infinity ($AUC_{0–inf}$) was calculated directly from the primary PK parameter estimates according to the following equation:

$$AUC_{0\text{-}inf} = \frac{Dose \times F}{CL}$$

where *F* is the bioavailability factor and *CL* is clearance. For each subject, $AUC_{0–inf}$ was calculated for a single dose in the amount received on Cycle 1 - Day 1 - Dose 1. Additionally, since the model incorporated between-occasion variability on bioavailability, separate $AUC_{0–inf}$ values were calculated using the bioavailability factors estimated for each occasion. Since between-subject variability could not be estimated reliably for CL and volume of distribution (V_d), all subjects were assumed to have the same typical value estimates for these parameters (see **Supplementary Table 2** and **Supplementary Table 3**). It is important to note that these findings do not imply that there is no between-subject variability in CL or V_d, but rather that the variability could not be estimated reliably due to the PK sampling times and the available number of subjects. The between-subject variability in the absorption rate constant reflects differences in the observed profiles between individuals. The between-occasion variability in bioavailability (F) impacts CL and Vd in a similar fashion as these parameters are actually CL/F and Vd/F for oral administration and captures the variability within individuals for nintedanib

.References

1. Beal SL. Ways to fit a PK model with some data below the quantification limit. J Pharmacokinet Pharmacodyn. 2001;28(5):481-504. Epub 2002/01/05. doi: 10.1023/a:1012299115260. PubMed PMID: 11768292.
2. Bergstrand M, Karlsson MO. Handling data below the limit of quantification in mixed effect models. AAPS J. 2009;11(2):371-80. Epub 2009/05/20. doi: 10.1208/s12248-009-9112-5. PubMed PMID: 19452283; PMCID: PMC2691472.
3. Mould DR, Upton RN. Basic concepts in population modeling, simulation, and model-based drug development-part 2: introduction to pharmacokinetic modeling methods. CPT Pharmacometrics Syst Pharmacol. 2013;2:e38. Epub 2013/07/28. doi: 10.1038/psp.2013.14. PubMed PMID: 23887688; PMCID: PMC3636497.

Supplementary Table 1: Pharmacokinetic Observations

Subject ID	Observations in model (*n*)	BLQ observations in model (*n*)
8013	8	0
7248	8	1
7755	8	1
7631[a]	8	0
8574	7	1
8241	8	4
7264	8	1
7340	8	0
6346	8	1
8287	8	0
7767	8	1
7687	6	0
6497	8	1
8804	8	0
6481[b]	5	1
6691	8	0
6086	8	1
7737	8	0
8263	8	1
6964[a]	8	1
8879	8	0
7449	8	0
6382[d]	8	1
7093	8	2
7521[c]	5	0
7352[d]	6	0
6838	8	0
6511[d]	8	0
8514	8	0
7165	8	1
**ALL**	229	19

BLQ below the lower limit of quantitation

[a] Subject did not return medication dosing diary

[b] Subject stopped treatment after two days

[c] Subject unevaluable

[d] Subject missed multiple doses

Supplementary Table 2: Primary Pharmacokinetic Parameter Estimates

Parameter description	Population estimate (%RSE of the estimate)	Between-subject-variability, as %CV (%RSE of the variability estimate)	Between-occasion variability, as %CV (%RSE of the variability estimate)
Typical value of first-order absorption rate constant (h^{-1})	0.635 (38%)	292 (12%)	NE
Typical value of bioavailability factor	1[a]	NE	57.9 (22%)
Typical value of apparent clearance (L/h)	555.5 (10%)	NE	NE
Typical value of apparent volume of distribution (L)	4631.1 (12%)	NE	NE
Residual unexplained variability – proportional, as %CV[b]	24.7 (19%)	NA	NA

CV coefficient of variation, *NA* not applicable, *NE* not estimated, *RSE* relative standard error

[a] Fixed to 1 as a reference. This does not imply that the typical value of bioavailability is actually 1, nor that the individual bioavailability values are near 1. This simply provides a reference point so that the bioavailability can vary across occasions.

[b] Over the typical range of observed concentrations (5–100 ng/mL), the combined additive and proportional residual unexplained variability ranges from 127.5–45.7 %CV.

Supplementary Table 3: Individual Primary Pharmacokinetic Parameter Estimates

Subject ID	Individual first-order absorption rate constant (h^{-1})	Bioavailability factor on Occasion 1[a]	Bioavailability factor on Occasion 2[a]	Apparent Clearance (L/h)[b]	Apparent Volume of distribution (L)[b]
8013	1.88	0.74	0.81	555.5	4631.1
7248	0.80	1.04	0.59	555.5	4631.1
7755	0.18	1.48	0.74	555.5	4631.1
7631	4.02	3.14	2.40	555.5	4631.1
8574	1.55	0.92	1.01	555.5	4631.1
8241	1.72	0.33	0.80	555.5	4631.1
7264	0.17	1.00	0.83	555.5	4631.1
7340	0.35	0.49	0.55	555.5	4631.1
6346	0.05	0.51	0.31	555.5	4631.1
8287	0.94	0.75	0.83	555.5	4631.1
7767	1.09	0.76	0.54	555.5	4631.1
7687	1.13	2.82	1.75	555.5	4631.1
6497	0.04	7.70	0.72	555.5	4631.1
8804	1.09	2.85	1.28	555.5	4631.1
6481[c]	0.15	0.33	-	555.5	4631.1
6691	3.34	0.32	1.57	555.5	4631.1
6086	1.38	1.47	1.15	555.5	4631.1
7737	0.66	1.82	1.73	555.5	4631.1
8263	0.16	0.65	0.43	555.5	4631.1
6964	1.45	1.56	1.87	555.5	4631.1
8879	0.12	1.10	0.61	555.5	4631.1
7449	0.08	1.55	1.63	555.5	4631.1
6382	2.25	0.12	0.65	555.5	4631.1
7093	0.05	1.93	1.16	555.5	4631.1
7521[d]	-	-	-	-	-
7352	0.47	0.49	0.31	555.5	4631.1
6838	0.28	2.26	0.47	555.5	4631.1
6511	1.27	0.51	0.80	555.5	4631.1
8514	0.18	1.64	1.69	555.5	4631.1
7165	0.87	0.48	0.40	555.5	4631.1

[a] Occasions 1 and 2 were defined as Cycle 1-Days 1–2 and Cycle 1-Day 15, respectively. *Note* that these values do not reflect actual bioavailability. The typical value was simply fixed at 1 to allow bioavailability to vary across occasions.

[b] Between-subject variability could not be reliably estimated for this parameter, therefore all individuals were considered to have the typical value estimate.

[c] Subject stopped treatment after two days.

[d] Subject unevaluable, only took morning doses, data not used in model fitting and parameter estimation.

Supplementary Table 4: Secondary Pharmacokinetic Parameters

Subject	Observed Values						Calculated Values[a]	
	Cycle 1 - Day 1 - Dose 1			Cycle 1 - Day 15 - Dose 1			$AUC_{0\text{-}inf}$ (ng×h/mL) Using bioavailability factor from occasion 1	$AUC_{0\text{-}inf}$ (ng×h/mL) Using bioavailability factor from occasion 2
	Dose (mg)	t_{max} (h)	C_{max} (ng/mL)	Dose (mg)	t_{max} (h)[b]	C_{max} (ng/mL)		
8013	200	0.63	29.2	200	1.98	23.0	268.07	291.83
7248	200	2.70	50.4	200	2.05	10.6	372.96	213.77
7755	200	7.00	30.5	200	2.48	26.9	532.89	268.19
7631[f]	150	0.97	190.0	150	3.55	76.6	849.10	648.60
8574	200	2.33	49.5	200	2.10	63.5	330.71	362.45
8241	200	NA	BLQ	200	2.00	53.1	120.21	289.53
7264	200	7.08	33.5	200	5.17	25.0	358.53	299.12
7340[c]	200	5.47	17.2	200	5.13	25.1	178.03	199.68
6346	200	7.10	5.4	200	2.18	11.0	184.20	111.79
8287	200	3.53	36.7	200	2.08	51.1	271.47	300.51
7767	200	2.00	35.1	200	5.00	11.8	274.20	193.04
7687	200	2.28	156.7	200	2.67	111.0	1013.97	631.00
6497	200	7.03	82.5	200	5.02	5.5	2771.31	259.02
8804	150	2.10	129.0	150	2.00	24.3	769.39	344.91
6481[d]	200	7.05	8.3	-	-	-	120.49	-
6691	200	0.75	18.1	200	2.45	25.3	115.60	565.94
6086[e]	200	5.05	41.4	200	2.03	75.2	528.24	311.07
7737	150	2.00	68.3	150	2.02	54.3	490.67	467.23
8263	200	5.03	16.8	200	2.88	39.1	234.34	155.27
6964[f]	200	2.38	85.8	200	2.18	108.2	560.61	672.69
8879	200	7.27	35.3	200	5.02	25.9	397.62	221.22
7449	200	5.05	26.7	200	5.07	91.1	559.14	588.30
6382[g]	200	2.10	5.6	200	2.03	47.3	41.68	232.84
7093	200	7.02	7.8	200	2.02	33.8	696.06	416.09
7521[h]	200	2.12	155.0	-	-	-	-	-
7352[f]	200	2.08	17.5	200	2.12	6.2	177.29	112.50
6838	200	2.08	55.6	200	5.03	15.7	814.11	169.12
6511	200	2.25	24.0	200	2.03	57.4	183.30	287.22
8514	200	5.40	48.7	200	5.28	82.5	589.70	608.14
7165	200	2.03	16.7	200	2.00	21.1	172.05	143.14

$AUC_{0\text{–}inf}$ area under the concentration–time curve from 0 to infinity, *BLQ* below the lower limit of quantitation, C_{max} maximum concentration, *NA* not applicable, t_{max} time at maximum concentration.

[a] Values were calculated from primary pharmacokinetic parameter estimates. For each subject and each bioavailability factor estimate, AUC_{0-inf} was calculated for a single dose in the amount received on Cycle 1-Day 1-Dose 1.

[b] Negative values indicate that the observed t_{max} for Cycle 1-Day 15-Dose 1 occurred in the pre-dose sample.

[c] Subject did not take any drug on Days 12–14.

[d] Subject stopped treatment after two days. Data excluded from summary statistics.

[e] Subject stopped taking drug on Days 3–11, resumed dosing on Day 12 at a reduced dose of 150 mg twice daily, and accidentally took a dose of 200 mg for Cycle 1-Day 15-Dose 1.

[f] Subject(s) did not return medication dosing diary or medication diary was found to be unreliable.

[g] Subject did not take any drug on Days 5–7.

[h] Subject unevaluable, only took morning doses, data not used in model fitting and parameter estimation. Data excluded from summary statistics.
